# Effects of different preservation on the mechanical properties of cortical bone under quasi-static and dynamic compression

**DOI:** 10.3389/fbioe.2023.1082254

**Published:** 2023-02-23

**Authors:** Jinlong Qiu, Zhikang Liao, Hongyi Xiang, Haocheng Li, Danfeng Yuan, Chengyue Jiang, Jingru Xie, Mingxin Qin, Kui Li, Hui Zhao

**Affiliations:** ^1^ Daping Hospital of Army Medical University, PLA, Chongqing, China; ^2^ Department of Medical Engineering, General Hospital of Central Theater Command, Wuhan, China; ^3^ School of Vehicle Engineering, Chongqing University of Technology, Chongqing, China; ^4^ College of Biomedical Engineering, Army Medical University, PLA, Chongqing, China

**Keywords:** cortical bone, mechanical properties, strain rate dependence, formalin fixation, bone dehydration, quasi-static and dynamic compression

## Abstract

**Introduction:** Mechanical properties of biological tissue are important for numerical simulations. Preservative treatments are necessary for disinfection and long-term storage when conducting biomechanical experimentation on materials. However, few studies have been focused on the effect of preservation on the mechanical properties of bone in a wide strain rate. The purpose of this study was to evaluate the influence of formalin and dehydration on the intrinsic mechanical properties of cortical bone from quasi-static to dynamic compression.

**Methods:** Cube specimens were prepared from pig femur and divided into three groups (fresh, formalin, and dehydration). All samples underwent static and dynamic compression at a strain rate from 10^−3^ s^−1^ to 10^3^ s^−1^. The ultimate stress, ultimate strain, elastic modulus, and strain-rate sensitivity exponent were calculated. A one-way ANOVA test was performed to determine if the preservation method showed significant differences in mechanical properties under at different strain rates. The morphology of the macroscopic and microscopic structure of bones was observed.

**Results:** The results show that ultimate stress and ultimate strain increased as the strain rate increased, while the elastic modulus decreased. Formalin fixation and dehydration did not affect elastic modulus significantly whereas significantly increased the ultimate strain and ultimate stress. The strain-rate sensitivity exponent was the highest in the fresh group, followed by the formalin group and dehydration group. Different fracture mechanisms were observed on the fractured surface, with fresh and preserved bone tending to fracture along the oblique direction, and dried bone tending to fracture along the axial direction.

**Discussion:** In conclusion, preservation with both formalin and dehydration showed an influence on mechanical properties. The influence of the preservation method on material properties should be fully considered in developing a numerical simulation model, especially for high strain rate simulation.

## 1 Introduction

Worldwide, more than nine million fractures occur every year, which results in prolonged disability and a huge economic burden on the healthcare system (Gribble et al., 2012). The most common causes of fractures are direct or indirect violence, such as falls, traffic accidents, military conflicts, etc., (Hedström et al., 2010; Chandler et al., 2017). Understanding the mechanical response of human bones is necessary to aid the improvement of both assessing injury severity and personal protection (Kumar and Ghosh, 2022). Numerical simulations are an effective tool to investigate the mechanisms of fracture from a microscopic perspective (Cronin et al., 2022; Hudyma et al., 2022). Numerous human finite element (FE) models, such as THUMS (Wu et al., 2017) and GHBMC (Park et al., 2016), have been utilized in impact biomechanics and medical studies. It is well known that the material properties are key factors affecting the bio-fidelity of FE models (Oftadeh et al., 2015). To obtain reasonable results from numerical simulations, the mechanical properties of cortical bone material must be clearly defined in the FE model (Asgharpour et al., 2014; Latella et al., 2015; Ramezanzadehkoldeh and Skallerud, 2017; Huang et al., 2018).

Mechanical testing of biomaterials is the most important way to improve the constitutive relationship and material properties of FE models (Sharir et al., 2008). Currently, bone samples used in biomechanical experiments are mainly preserved by fresh freezing, storage in chemical reagents such as formalin, and dehydration (Currey, 1988a; van Haaren et al., 2008; Unger et al., 2010). Fresh freezing samples resemble the *in vivo* situation most accurately. While, it is difficult to obtain abundant supply of fresh frozen human bone, especially for young population. Besides, freezing carries a higher preservation cost and increases the risk of infection (Demiryürek et al., 2002). Therefore, formalin-fixed and dehydrated samples are still widely used for understanding of biomechanical properties. Cortical bone is made of mineralized collagen fibrils, lamellae, osteon and haversian systems correspondingly (Sabet et al., 2016). The mechanical behavior of cortical bone is determined by the material composition and structural arrangement. However, formalin fixation or dehydration not only affects the organic matter in the bone but also the inorganic matter and therefore alters the mechanical properties of bone (Kikugawa and Asaka, 2004). Therefore, it is of great significance to accurately understand the effect of preservation on the mechanical properties of bone.

Many studies have investigated the effect of formalin preservation on the mechanical properties of bone, but there are some discrepancies and disputes. Some researchers stated that preservation influences the mechanical properties of bone (Goh et al., 1989; Ohman et al., 2008; Burkhart et al., 2010; Morita et al., 2013; Wieding et al., 2015), while others believe that formalin fixed less than 12 months did not significantly change the mechanical properties of the bone (van Haaren et al., 2008; Nazarian et al., 2009; Topp et al., 2012). This is partly due to the different usage of bone, solution, preservation time and loading method. Therefore, it is difficult to compare these experimental results and draw conclusions to guide the future optimization of the FE model. The effects of dehydration have been preliminary reported in literature, whereby with an increase of water loss, dried bone shows increasing strength and stiffness and decreasing toughness due to water-mineral interaction removal (Nyman et al., 2006; Hoffseth et al., 2017). In addition, it is important to emphasize that most of the literature was conducted by quasi-static loading. However, fractures can be divided into low-energy fractures and high-energy fractures (Diamantopoulos et al., 2012). Low-energy fractures are associated with events such as falls from a standing height and occur mainly in the elderly population because of osteoporosis. On the other hand, high-energy fractures are associated with high-velocity loading events, such as motor vehicle crashes and military blast scenarios. Impact energy is regarded as the main difference between these injury environments and distinctly influences the mechanical properties of bones. The mechanism of fracture injury varies under different impact states (Mayeur et al., 2013; Wallace et al., 2013; Gauthier et al., 2017). It has been revealed that strain rate has a great influence on the mechanical properties of cortical bone. Previous studies have demonstrated that the mechanical properties of biological tissue are strain rate dependent (Lei et al., 2020). To the best of our knowledge, the effect of preservation on the mechanical properties of bone in a wide strain rate range has not been studied.

It is hypothesized that the mechanical properties of cortical bone, especially the strain rate dependence, may be influenced by different preservation methods. In this study, quasi-static and dynamic compression were performed on machined cuboid-shaped samples of cortical bone from pig femurs. Material parameters were then calculated to evaluate the alteration in the elastic and plastic behavior of cortical bone due to microstructure changes caused by formalin fixation and dehydration. This study will play a key role in understanding the gap of bone mechanical property from different preservation method among wide strain rates, which is necessary to configure the material property of FE models.

## 2 Methods

### 2.1 Sample preparation

Porcine femurs, which have similar hierarchical structures to human femurs (Kieser et al., 2014) and are convenient to collect, were bought from a single abattoir (Wu et al., 2012). Ethics committee of Daping Hospital did not require the study to be approved by an ethics committee because the used samples were by-product of routine industry. All tested pigs were of the same species, fed in the same way and slaughtered at age 6–7 months. The femurs were processed into regular samples using a self-designed tool under a saline rinse. Processing consisted of two main procedures. First, the position of the femur was adjusted to ensure that the plane of the circular saw was vertical to the bone axial. Bone was cut along the same diaphyseal cross-section by avoiding regions of ligament connection (Nobakhti et al., 2017). Second, radical, and circular dimensions were machined based on the transverse planes acquired regardless of radical anatomical positions (Abdel-Wahab et al., 2011; Ferreno et al., 2017; Mansor et al., 2017). In order to uniformly distribute the stress on the samples under static compression, the ratio of longitudinal size to transversal size was designed as 2, whereas the ratio was one during dynamic compression to achieve stress equilibrium quickly. The samples were machined into 4 mm × 4 mm × 8 mm cubes for static tests and 4 mm × 4 mm × 4 mm cubes for dynamic tests.

### 2.2 Experimental group

A total of 96 regular samples were derived and divided into three groups of fresh, formalin-fixed, and dehydration dried, which are listed in Table 1. There were eight samples per group at a specific strain rate. The fresh group was instantly tested. The dried group was stored in a vacuum drying chamber at 80°C for 48 h to partly remove water and weaken the collagen phase. The formalin-fixed group was stored in 4% formalin solution for 6 weeks. All specimens were tested within 6 h after preservation was accomplished.

**TABLE 1:** Sample number of each group.

Strain rates	10^−3^ s^−1^	10^−2^ s^−1^	10^2^ s^−1^	10^3^ s^−1^
Fresh	8	8	8	8
Formalin	8	8	8	8
Dehydration	8	8	8	8

### 2.3 Compression testing

Static compression tests (strain rate at 10^−3^ s^−1^ and 10^−2^ s^−1^) were conducted on an electronic material testing machine (Instron^®^ Model 5,969; Instron, United States) with a load cell capacity of 10 kN. Loading rates were calculated according to the longitudinal length of each specimen and strain rate. All tests were performed at room temperature with occasional sprinkling of saline to keep the samples hydrated. The contact surface between the sample and the platen was lubricated with petroleum jelly, and it was confirmed that the two loading surfaces of the sample were parallel and no horizontal displacement occurred during the compression process.

**FIGURE 1:**
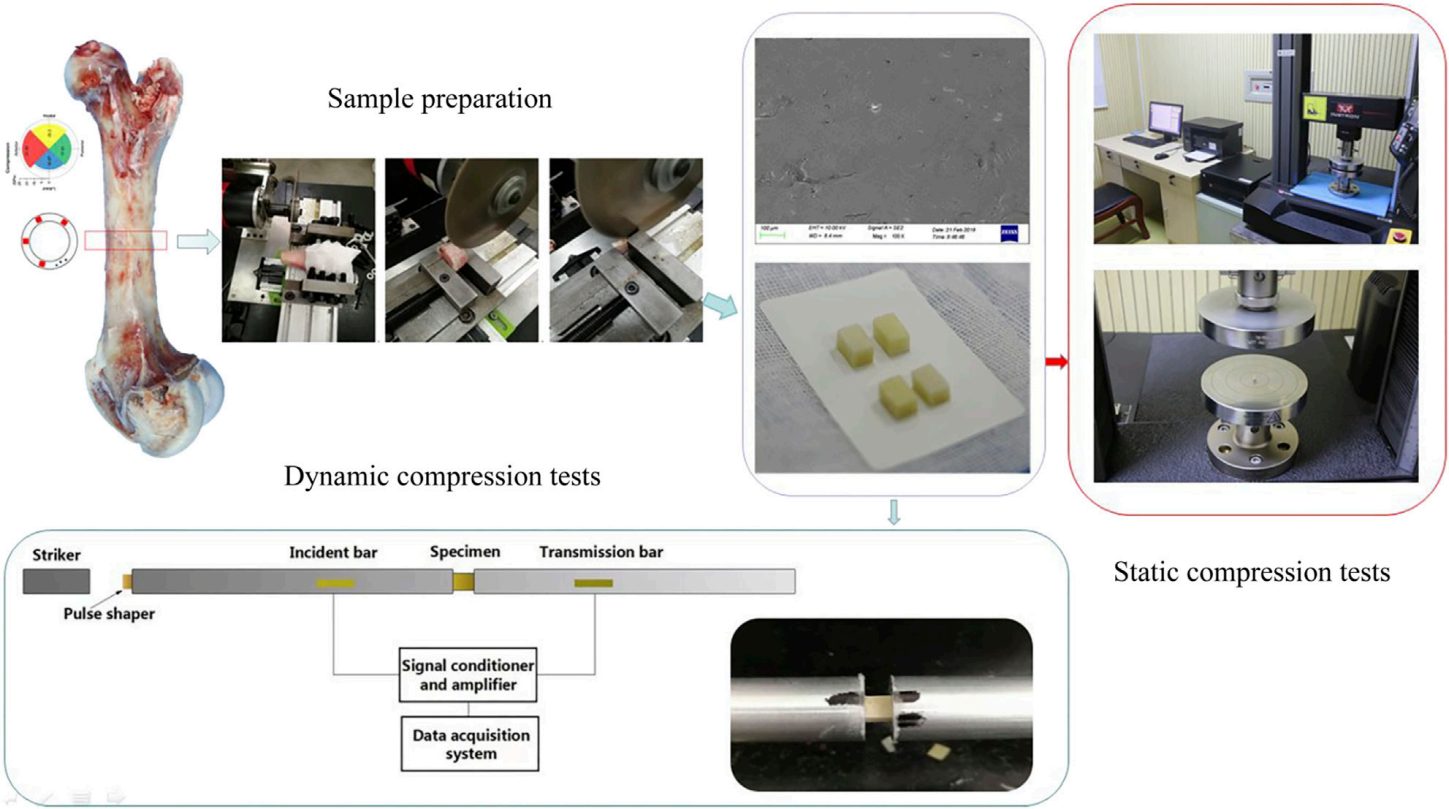
Schematic diagram of the experimental procedure.

**FIGURE 2:**
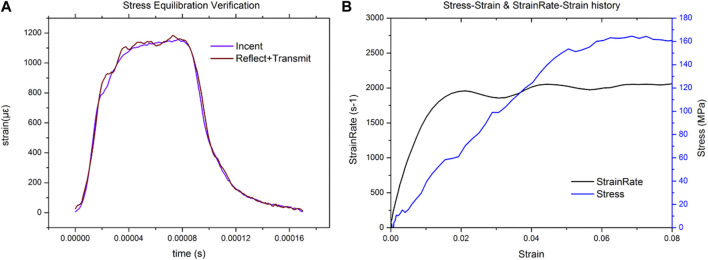
Representative plot for the dynamic equilibrium in the sample during the SHPB experiment **(A)**, representative strain signal obtained from the SHPB test **(B)**.

Dynamic compression tests of bone sample were performed by a modified Split Hopkinson Bar (SHPB). The striker bar, incident bar, and transmitter bar have the same diameter of 14.5 mm and different lengths of 200 mm, 1,500 mm, and 1,500 mm (Aluminum 7A04 bars). The bone sample was sandwiched between the incident bar and the transmitter bar. Both semiconductor strain gauges (120 Ω, GmbH, Germany) across the bar diameter at a bar location were connected in series to the same leg in the Wheatstone bridge to offset the influence of bending stress. The strain gauges were connected to a signal conditioner amplifier and data was recorded with a Dynamic Signal Acquisition and Analysis System (Donghua, China) at a sampling rate of 1 MHz. In order to achieve stress equilibrium and constant strain rate conditions, a pulse shaper processed from paper jam into a uniform cylinder was placed between the striker bar and the incident bar. According to theory of the one-dimensional wave transmission, the strain rate increases with the diameter of the pulse shaper. Similarly, the slope of the loading wave increases with the striker speed and loading time. Thus, a uniformly machined copper cylinder pulse shaper was used at different sizes for filtering high frequency waves. A schematic diagram of the experimental procedure is shown in Figure 1. The representative curves obtained are shown in Figure 2A. Over the entire pulse duration, the signal is consistent with Eq. (1). Therefore, it can be considered that the stress equilibrium is maintained in the experiment.

$$\varepsilon_{I}+\varepsilon_{R}=\varepsilon_{T}$$ (1)

where $\varepsilon_{I}$ is the incident wave, $\varepsilon_{R}$ is the reflected wave and $\varepsilon_{T}$ is the transmitted wave. The stress-strain curve calculated from the pulse signal is shown in Figure 2B. The strain rate rises rapidly to the expected constant strain rate, and then remains constant until specimen failure. The strain occurring in the specimen at a constant strain rate condition exceeds 70% of the total strain. Therefore, the method designed in this paper is effective in reflecting the compressive mechanical properties of cortical bone at the preset strain rate.

### 2.4 Fragment morphology

Macro images of compressed fragments were taken by an optical camera (EOS 70D, Canon, Japan). Micro images of compressed fragments were taken using scanning electron microscopy (SEM, Sigma300, Zeiss, German). The SEM images were used to examine the fracture surfaces, and to explain the effects of structural deformation and failure in both fracture toughness and bending tests. A matter of concern in dynamic compression tests compared with fracture toughness tests is that the cracks may initiate and connect before propagating a long distance, which is closer to fracture accrued by a suddenly applied load on most occasions. Thus, it was essential to focus on the morphology of the fragments of tested samples.

### 2.5 Statistical analysis

Three mechanical parameters (ultimate stress, ultimate strain, and elastic modulus) calculated from experimental testing data were analyzed using statistical software (SPSS, version 20). Ultimate stress is considered as the maximum stress in the stress-strain curve, whereas ultimate strain is considered as the strain at peak stress. The elastic modulus describes the tendency of a material to undergo elastic strain when subjected to stress and can be determined by the slope of the stress-strain curve. A one-way ANOVA test was performed to determine if the preservation method showed significant differences in mechanical properties at different strain rates. *p*-values of *p* < 0.05 were considered statistically significant.

## 3 Results

### 3.1 Compressive properties of each group

**FIGURE 3:**
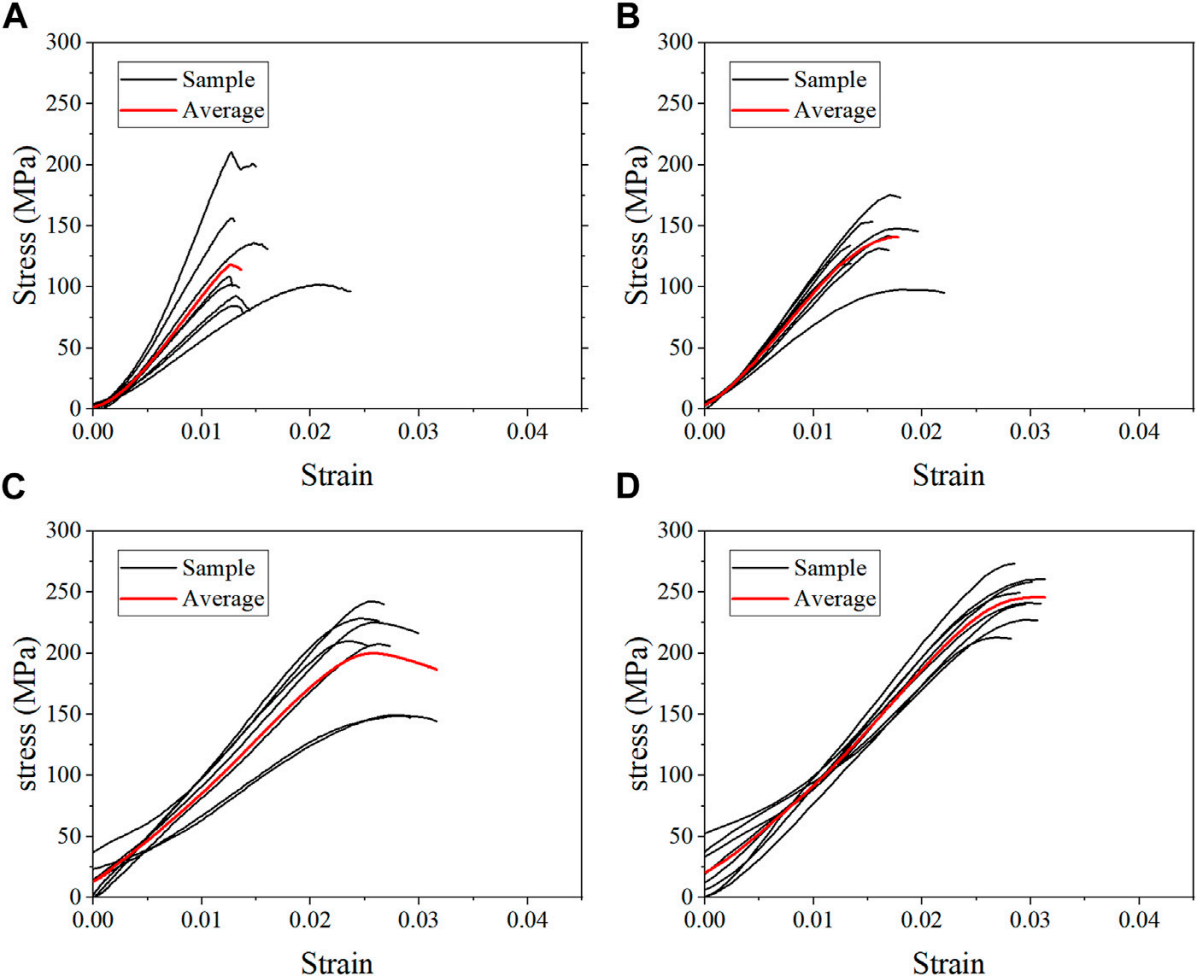
Average stress-strain curves for the fresh group at strain rate 10^−3^ s^−1^ **(A)**, 10^−2^ s^−1^ **(B)**, 10^2^ s^−1^ **(C)**, and 10^3^ s^−1^ **(D)**.

**FIGURE 4:**
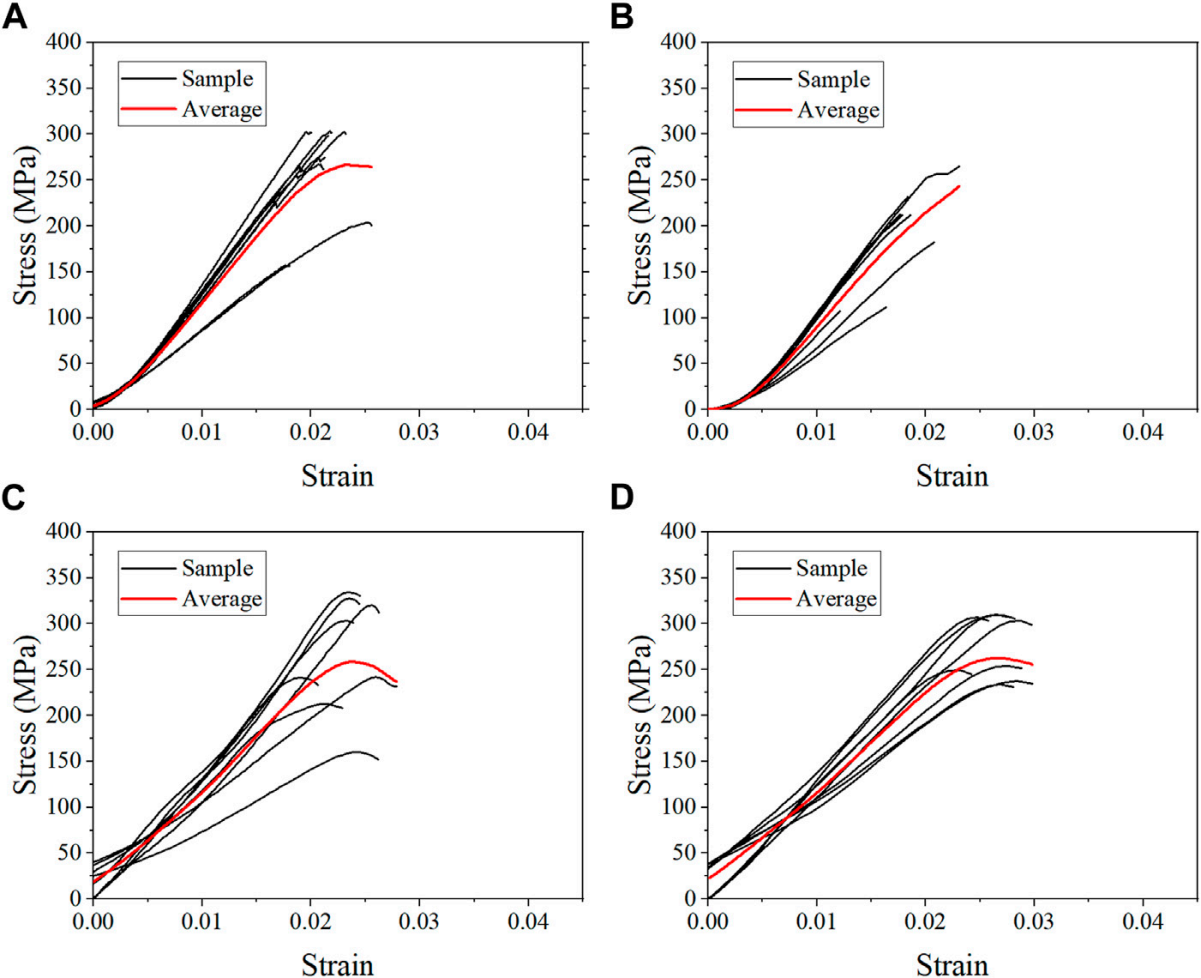
Average stress-strain curves for the formalin group at strain rate 10^−3^ s^−1^ **(A)**, 10^−2^ s^−1^ **(B)**, 10^2^ s^−1^ **(C)**, and 10^3^ s^−1^ **(D)**.

**FIGURE 5:**
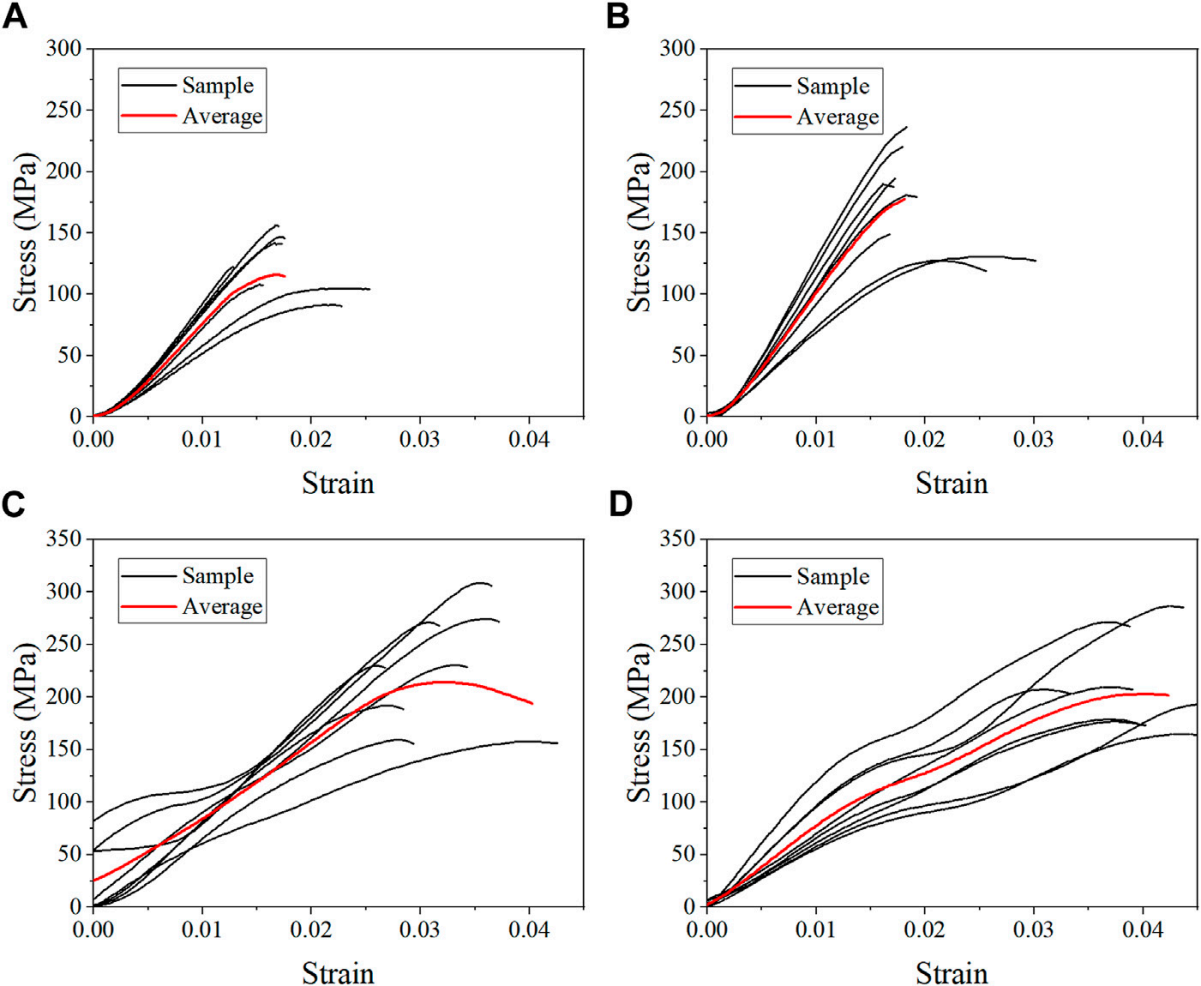
Average stress-strain curves for the dehydration group at strain rate 10^−3^ s^−1^ **(A)**, 10^−2^ s^−1^ **(B)**, 10^2^ s^−1^ **(C)**, and 10^3^ s^−1^ **(D)**.

The average stress-strain curves for the fresh, formalin and dehydration groups were shown in Figures 3, 4, 5 respectively. All curves both include elastic and plastic deformation phases. The stress and strain are essentially linear at the beginning of the loading phase indicating that the bone underwent elastic deformation during this stage. As the stress continues to increase, the slope of the curve decreases showing a non-linear relationship, indicating that plastic deformation occurs. The difference was that most of the samples in the quasi-static group fracture within a very small strain after yielding. On the contrary, the elastic and plastic phases of bone were prolonged under dynamic loading. Based on the stress-strain curve, the elastic modulus, ultimate stress, and ultimate strain were calculated for each group. As shown in Table 2, the value of the elastic modulus decreases for almost all groups with the increase in strain rate except for formalin group at 10^−2^ s^−1^. Ultimate stress and ultimate strain increase with the strain rate for the fresh and formalin groups. The value of ultimate stress increased by 80.2%, 47.7% and 1.8% for fresh, formalin and dehydration group samples, respectively, when the strain rate varied from 10^−3^ s^−1^ to 10^3^ s^−1^. On the other hand, the percentage increment in ultimate strain was 47.2%, 24.4% and 29.2% for fresh, formalin and dehydration group samples, respectively. The value of the elastic modulus decreased by 31.7%, 15.2% and 23.9% for each group when the strain rate varied from 10^−3^ to 10^3^ s^−1^.

**TABLE 2:** Mechanical properties obtained from compression tests at different strain rates.

Group	Strain rate (s^−1^)	Modulus (GPa)	Stress (MPa)	Strain
Fresh	10^−3^	11.42992	111.64078	0.01345
	10^–2^	10.27757	137.64466	0.01708
	10^2^	8.51293	192.15465^a^	0.02699##
	10^3^	7.8123	201.15271^a^	0.03325##
Formalin	10^–3^	9.37669	124.43589	0.01833
	10^–2^	12.07201	178.56487^a^ ^,^ ^b^	0.02027
	10^2^	8.58512	227.95492^a^	0.03336##
	10^3^	8.04376	228.09941^##^	0.04114^##^ ^b^
Dehydration	10^–3^	14.24331	263.6619**	0.02198**
	10^–2^	13.48866^b^	191.64064^a^ ^,^ ^b^	0.01812^a^
	10^2^	13.0629**	267.58501**	0.0246
	10^3^	10.83874^a^ ^,^ ^b^	268.51274^b^	0.02839^##^ ^b^

^a^ Significant difference with respect to the strain rate of 10−3s−1 within the group, ^#^<0.05, ^##^<0.01.

^b^ Significant difference to the control group at the corresponding strain rate, *<0.05, **<0.01.

### 3.2 Strain rate sensitivity of each group

**FIGURE 6:**
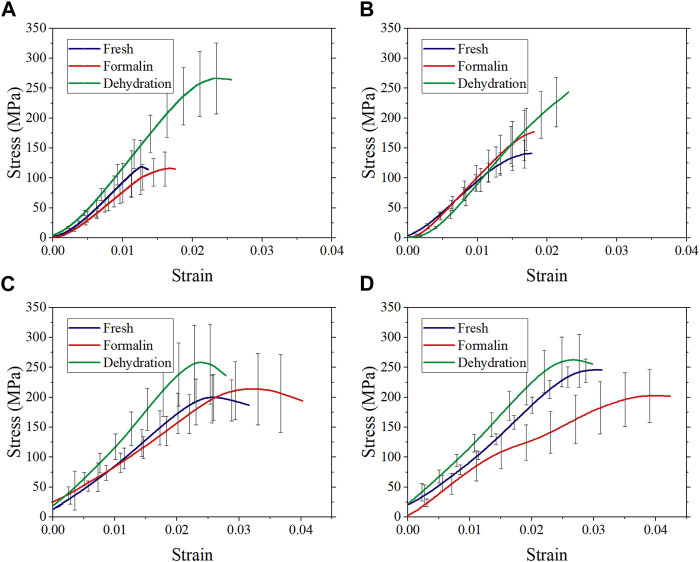
Average stress-strain curves with standard deviation at strain rate 10^−3^ s^−1^ **(A)**, 10^−2^ s^−1^ **(B)**, 10^2^ s^−1^ **(C)**, and 10^3^ s^−1^ **(D)**.

**FIGURE 7:**
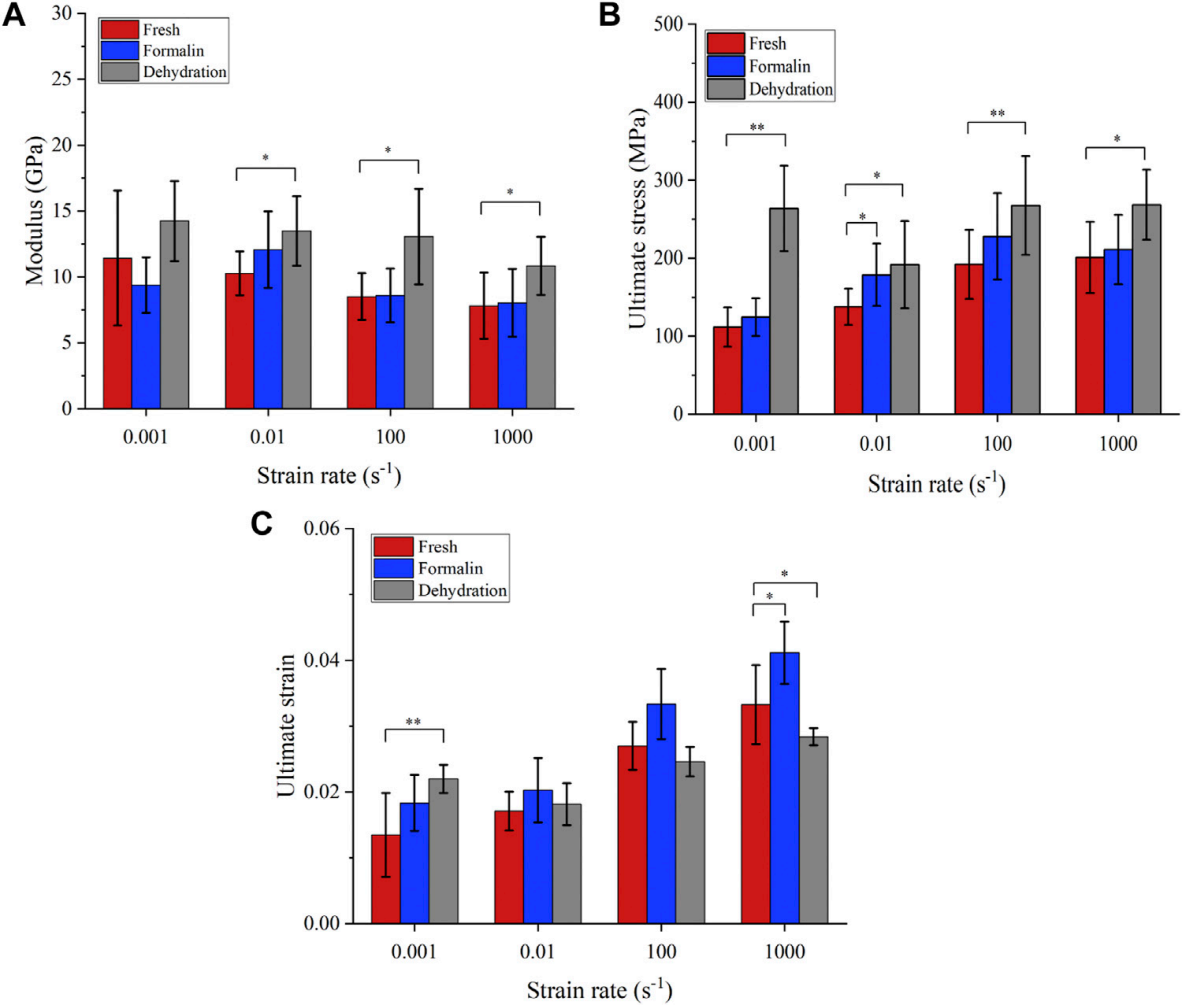
Mechanical properties obtained from compression tests. Elastic modulus **(A)**, failure stress **(B)**, and failure strain **(C)**. (*p-value <0.05, **p-value <0.01).

The average stress-strain curves with standard deviation are shown in Figure 6. Preliminary observation showed that there were differences in the stress-strain curves between different groups. Figure 7 shows that the elastic modulus of the dehydration group was higher than that of the other two groups. There was a statistical significance between the dehydration group and the fresh group at a strain rate of 10^−2^, 10^2^, and 10^3^ s^−1^. The ultimate strain and stress were higher in the formalin and dehydration groups compared to the fresh group.

In order to further investigate the strain-rate dependent behavior of cortical bone, the strain-rate sensitivity exponent (m) was calculated. For a given strain and temperature, the strain rate sensitivity is given as Eq. (2):

$$m=\frac{∆\log\sigma }{∆\log\dot{\varepsilon }}|\varepsilon ,T$$ (2)

where m is the strain rate sensitivity exponent, *σ* is stress and *•ε* is the applied strain rate. The value of m can be calculated from the log-log plot of stress and strain-rate. A similar approach has been used to calculate the value of m for biomaterials in previous studies (Gao et al., 2016; Zhao et al., 2017).

**FIGURE 8:**
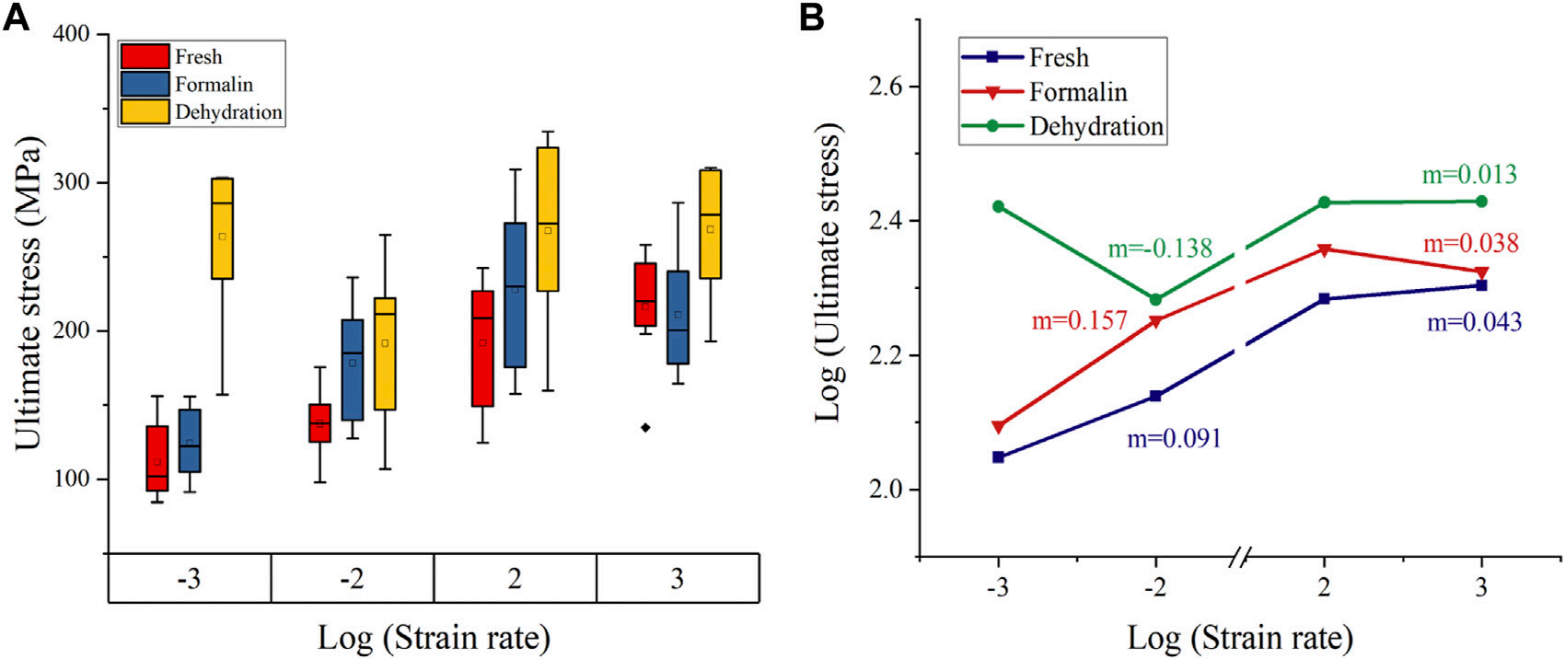
Failure stress variation with strain rate **(A)**. Double logarithmic plot for stress-strain rate **(B)**. Strain rate sensitivity exponent m was calculated from the slope of the curve (The base of the logarithm was ten).

Figure 8 shows the ultimate stress-strain rate on double logarithmic scales. The value of ultimate stress increases with the strain rate for all groups except for the dehydration group at a 10^-2^s^-1^ strain rate and the formalin group at a 10^-3^s^−1^ strain rate. For the fresh and formalin group, the value of m was found to be higher within quasi-static strain rates (*m* = 0.091–0.157) as compared to high strain rates (*m* = 0.038–0.043). For strain rates lower than 10^−2^s^−1^, the value of the strain rate sensitivity exponent m was highest for the fresh group, whereas dry-treated bones showed the lowest m at high strain rates.

### 3.3 Microstructure of cross-sections

**FIGURE 9:**
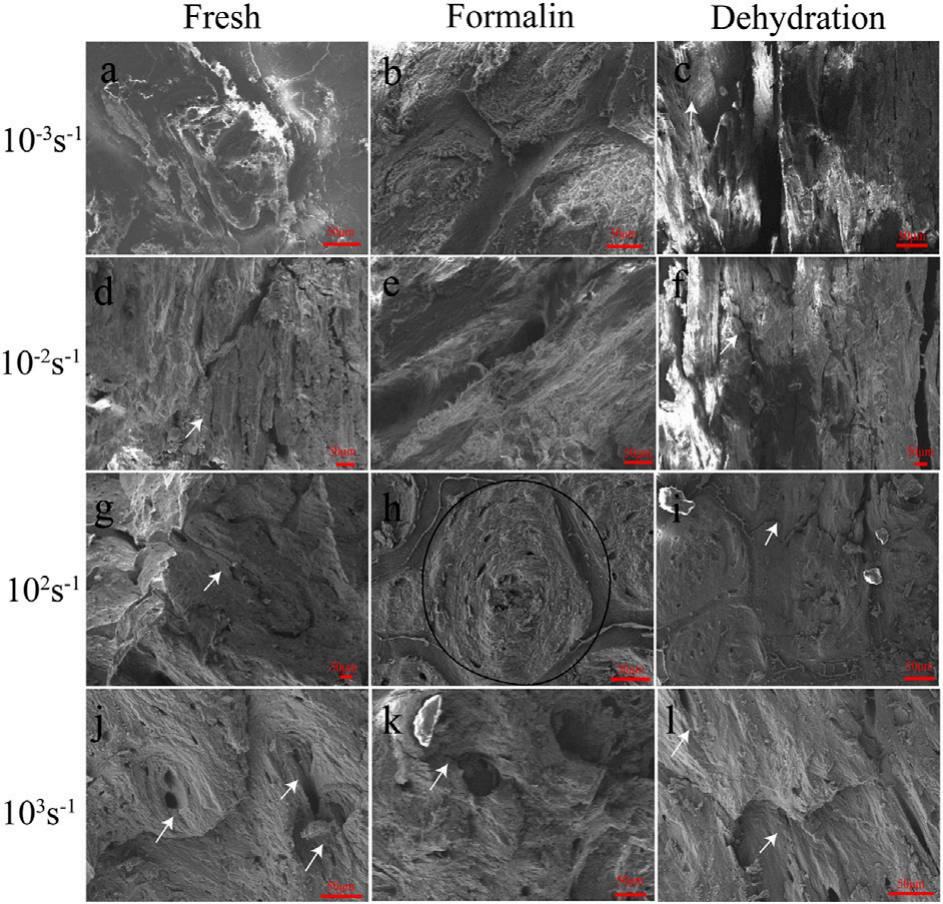
SEM images of fresh **(A, D, G, J)**, formalin **(B, E, H, K)**, and dehydration **(C, F, I, L)** samples after compression for strain rates of 10^−3^ s^−1^ **(A, B, C)**, 10^−2^ s^−1^ **(D, E, F)**, 10^2^ s^−1^ **(G, H, I)** and 10^3^ s^−1^ **(J, K, L)**. Bellow the samples the according magnifications are presented. The white arrows indicate cracks. The region within the black ellipse was considered to be a region of the Havers’ canals.

This study focused on photographing the characteristics of Havers’ canals and their surrounding structures, as shown in Figure 9. To minimize bias in the selection of images, samples with the ultimate stress closest to the mean ultimate stress for the given strain rate were chosen. The typical structure of Havers’ canal was shown in the black ellipse in Figure 9H. The representative shear cracks were indicated by black arrows. The microscopic morphology of sections in the static compression group shows that drying and formalin significantly changed the interaction of the cortical bone microstructure. The concentric lamellar bone structures around the Havers’ canals were clearly observed, and the crack surface was at a certain angle to these junctions (Figure 9A). As the strain rate increases, the Havers’ canals and lamellae on the crack surface were seriously damaged, and the shear band was visible in Figure 9D. The lamellar bone surrounding the fracture surface of the preserved cortical bone had the same concentric alignment as the fresh bone. However, lamellar bone pulled out by shear stress during dehiscence tends to form more curved edges with fragments, as shown in Figures 9B,E. On the fracture surface of dried cortical bone, an intermittent exposure of Havers’ canals was observed. In addition, the lamellar bone was barely pulled out, leaving only white spots on the broken surface that appeared to be collagen fibers, as showed in Figures 9C, F.

The fracture surface caused by dynamic compression is also significantly different from that caused by static compression. At a strain rate of 10^2^ s^−1^–10^3^ s^−1^, the roughness of the fracture plane of fresh cortical bone was significantly lower than that of the static plane, and the effect of shear stress on the lamellae was only reflected in the protruded processes at local locations in the plane (Figures 9G,J). The lamellar structure of the formalin-fixed cortical bone became less obvious and was pulled out in the form of fibers (Figures 9H,K). As the strain rate increased, the surface of the bone showed obvious shear stress and matrix deformation in the same direction. The fracture direction of the dehydration bone was similar to that of static compression, but there were transverse lines with a small spacing in the direction perpendicular to the fracture surface (Figures 9I, L).

### 3.4 Macroscopic fracture morphology

**FIGURE 10:**
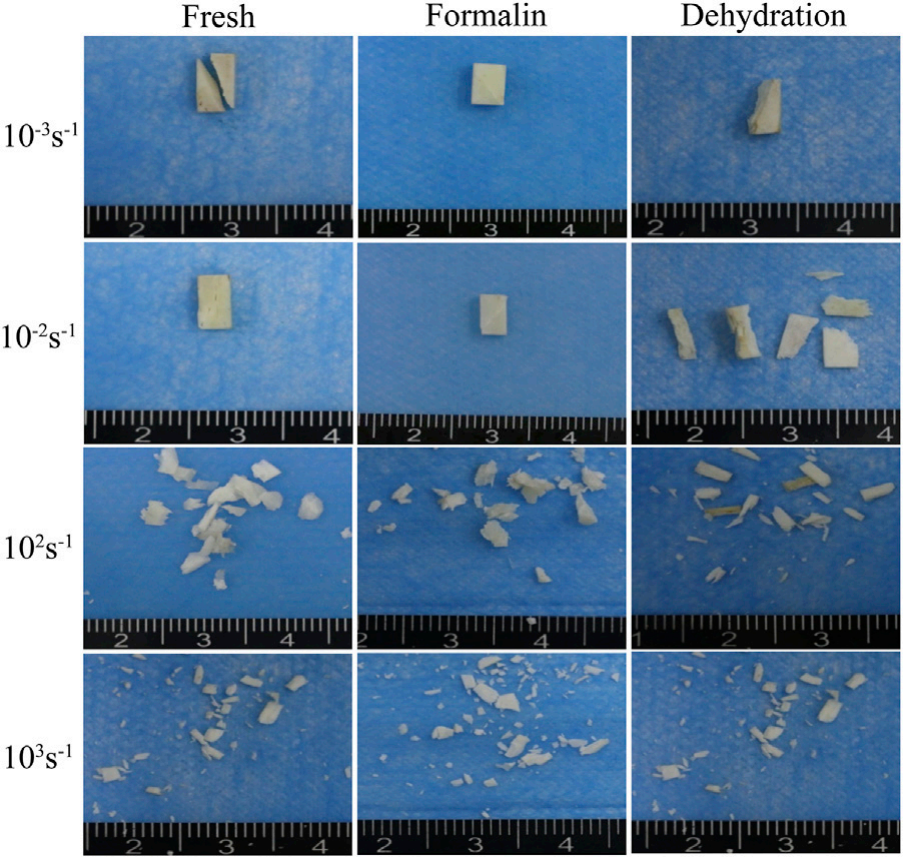
Macroscopic fracture morphology of fresh **(A)**, formalin **(B)**, and dehydration group **(C)** samples taken by an optical camera.

Typical fracture fragments of each sample were shown in Figure 10. In the static compression test (strain rates 10^−3^ s^−1^ and 10^−2^ s^−1^), the fresh bone, dried bone, and formalin-fixed bone showed the same fracture morphology. The angle between the fracture surface and the bone axis was approximately 30°. As the strain rate increased from 10^−2^ s^−1^ to 10^3^ s^−1^, the fragment sizes of the two groups gradually decreased. The formalin group and the fresh group had similar fracture angles, and the cracks always expanded along the oblique direction. However, the fracture surface of the dried group showed a smaller angle relative to the bone axis direction, which was different in direction to the other groups. In addition, the specimen was broken into fragments in the dynamic compression test and the size of fragments decreased with the increase of strain rate.

## 4 Discussion

Cortical bone exhibits typical elastic-brittle mechanical responses both under static and dynamic compression in this study. The standard linear elastic response is observed by maintaining a constant strain rate through a modified Hopkinson bar. The key to maintaining constant strain rate for elastic brittle materials such as bone tissue is that the slope of incident pulse plateau is equal to that of transmitted pulse. Comparing with cutting edge material shaping method in previous studies (Nyman et al., 2006; Burkhart et al., 2010; Sanborn et al., 2016) the wave shaping method established in this research is more stable in achieving constant strain rates.

**FIGURE 11:**
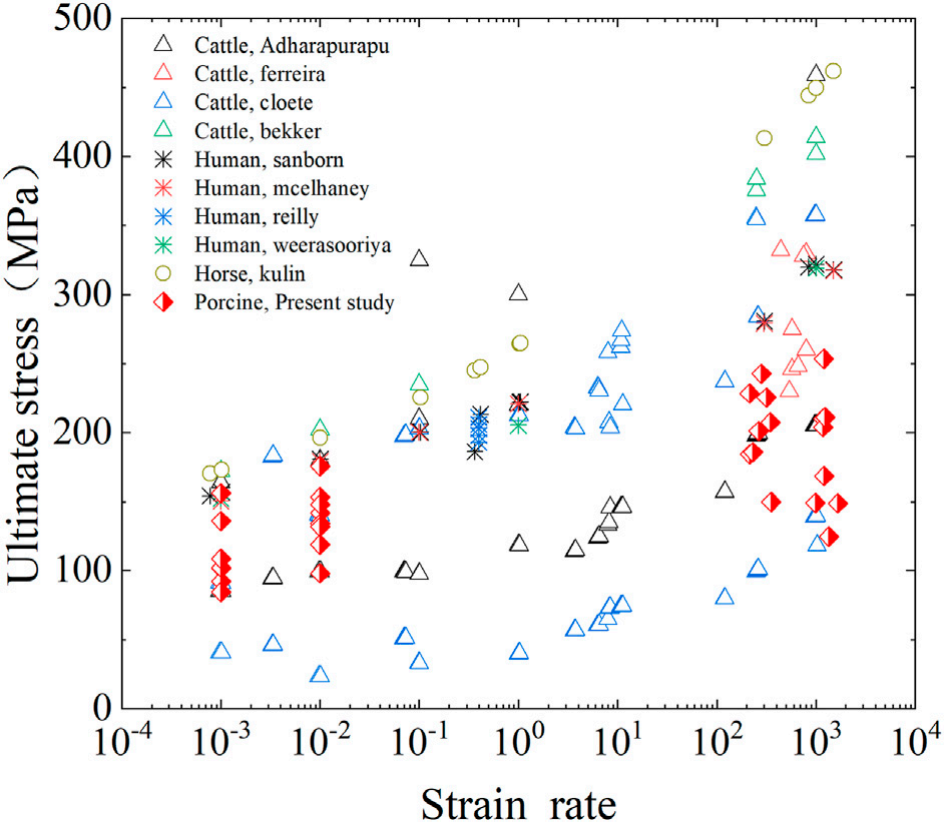
Comparison of ultimate stress of cortical bone in fresh group with previous studies.

The ultimate stress of fresh cortical bone was compared with data reported in previous literatures. As shown in Figure 11, the published femur compression tests include human, bovine, horse, and pig bones. Although the experimental method and sample species are inconsistent, the ultimate stress of cortical bone in human and the other three animals showed an increasing trend with the increase of strain rate. The cortical bone of horse femur has the largest ultimate stress, followed by human femur and pig femur. Bovine femur, the most used in previous studies, has the largest difference in ultimate stress results. The results present in this study were consistent with most of the results in terms of parameter values and trends, which validates the accuracy of the established method for testing the compression behavior of cortical bone.

In terms of the mechanical properties of cortical bone, with the strain rate increasing from 10^−3^ s^−1^ to 10^3^ s^−1^, the ultimate stress and ultimate strain of fresh bone increased by 80.2% and 47.7%, respectively, and elastic modulus decreased by 31.7% (Currey, 1975). Also indicated that increasing strain rate increases yield strength, tensile strength, the value of strain at yield, but the elastic modulus was unvarying over physiological strain rates (Currey, 1988b). Similarly (Ferreira et al., 2006) tested the mechanical properties of bovine cortical bone at high strain rate and stated that for both longitudinal and transverse directions the elastic modulus decreased and the ultimate strength increased for higher strain rates. However (Johnson et al., 2010) demonstrated with initial modulus increasing by more than a factor of 2 as applied strain rate is increased from 0.001 to 1500 s^−1^. Therefore, the effect of strain rate on the elastic modulus is still somewhat controversial.

The ultimate stress of formalin-fixed samples increased by 47.2%, and the ultimate strain increased by 24.4% 10^−3^ s^−1^–10^3^ s^−1^. The ultimate strain and ultimate stress of dried bone increased by 1.8% and 29.2%, respectively. However, not all values exactly match this variation, including elastic modulus for formalin group at 10^−2^ s^−1^, ultimate strain and ultimate stress for dehydration group at 10^−2^ s^−1^. This could be the effect of formalin and dehydration on the bone sample itself. On the other hand, these abnormal values may be due to individual differences in the pigs or to the fact that the samples were not prepared in a completely consistent direction. Because of the anisotropy of biomaterials, there biomechanical properties differ in the longitudinal and transverse directions (Szabo and Rimnac, 2022; Manandhar et al., 2023). In addition, these influences also contribute to the larger variance of mechanical parameters. Overall, ultimate stress and ultimate strain increased as the strain rate increased, while the elastic modulus decreased. The strain-rate sensitivity exponent for the fresh, formalin-fixed and dehydration samples were 0.043, 0.038, and 0.013, respectively. The strain rate sensitivity values calculated in this study were within the range of previous studies on cortical bone (Carter and Hayes, 1976; Peruzzi et al., 2021). Although the effects of formalin preservation and dehydration preservation on the ultimate stress, ultimate strain and elastic modulus are debatable, these preservation methods significantly reduced the strain rate dependence of cortical bone compared with fresh bone.

The possible reasons for the change of mechanical properties were that formalin and dehydration affect the connection between collagen fibers and bone matrix. Bone mainly consists of an organic matrix, mineral reinforcement and water (Novitskaya et al., 2011). Formalin affects the inorganic and organic components of bone (Kikugawa and Asaka, 2004). Ca, P, Mg and other elements in the hydroxyapatite of bone dissolve in acidic formalin solution (Boskey et al., 1982), which results in decreased bone mineral content and increased porosity (Turner, 2002). There were several unevenly distributed cracks in the fresh cortical bone section, which sprouted at various locations in an oblique direction under positive and tangential stresses. Soaked in formalin increased the ductility of the collagen and lamellae, furthermore, weaken the lamellar strength because of the alteration of the collagen linkage in the cortical bone (Nyman et al., 2006). Propose a model for the effects of water distribution on mechanical behavior of bone as follows. The water bound to the collagen fibrils provides post-yield toughness to bone. Removing this water increases strength and stiffness.

**FIGURE 12:**
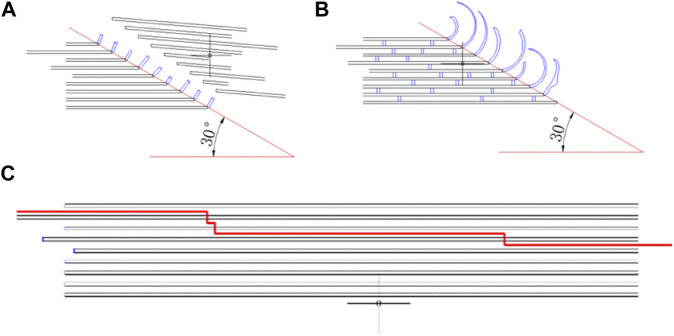
Mechanism of lamellar fracture for fresh **(A)**, formalin **(B)**, and dehydration group **(C)**.

The fracture procession of the four strain rate groups can be divided into three stages with the increase of strain rate. The first stage is static compression. In this stage, the lower strain rate enables the interaction between the microstructures to fully play out, resulting in the coarsest section due to a greater plastic deformation of the microstructures. The fracture angle of dried bone also verified the difference in collagen connection between dried and fresh bone (Figure 12). The second stage is dynamic compression between 10^2^ s^−1^ and 10^3^ s^−1^. In this stage, a very flat fracture surface was formed as the plastic deformation on the fracture surface decreases and the rate of crack extension increases. The result was consistent with that of Adharapurapu et al. (2006). The formation and confluence of multiple cracks, rather than the expansion of a single crack, become the main mechanism of specimen failure (Lin et al., 2016). The study of fracture toughness of single-precrack or double-precrack specimens in the dynamic range of the bone fracture mechanism may not be able to simulate the dynamic fracture process of bones (Nobakhti et al., 2017); (Kieser et al., 2014) also confirmed this point for the compression and fracture toughness of equine cortical bone. This may be related to the different stress distribution and deformation pattern between bending and compression tests. The third stage is when the strain rate is greater than 10^3^s^-1^. In this strain rate range, the surface of the three cortical bone fragments shows a wavy shape, which has not previously been observed in the literature. The results indicated that there may be an extrusion between the fragments at the same time of fracture.

## 5 Conclusion

This study shed light on the effects of different preservation methods on the strain rate-dependent mechanical response of cortical bone from quasi-static to dynamic compression. The results show that ultimate stress and ultimate strain increased as the strain rate increased, while the elastic modulus decreased. Formalin fixation and dehydration did not affect elastic modulus significantly whereas significantly increased the ultimate strain and ultimate stress. The strain rate sensitivity of formalin-fixed and dried bones reduces at high strain rates. Formalin fixation and dehydration treatments induced a connection of collagen in the bone, causing a significant alteration in macro- and microscale mechanical properties. Both formalin immersion and dehydration will affect the bone material mechanical properties, especially at high strain rate compression. The results of the present study can be helpful in establishing an accurate numerical model and further investigating the mechanism of bone trauma at high strain rates.

## 6 Limitations

Despite outstanding results presented here, some shortcomings existed for the current study. Firstly, to get dehydrated bone rapidly, the bone samples were kept in a drying chamber at 80°C for 48 h. However, the fact is that the bone samples for material mechanical test will not be preserved at the aforementioned temperature. Secondly, compression tests on cortical bone with moderate strain rates were not performed due to the limitations of the testing machine. Thirdly, the bone tissue samples in this study were obtained from pig femurs rather than human specimens, and differences in material properties between species were not considered.

## Data Availability

The original contributions presented in the study are included in the article/Supplementary Material, further inquiries can be directed to the corresponding authors.

## Abbreviations

FE, Finite Element; SHPB, Split Hopkinson Bar; SEM, Scanning Electron Microscopy.
